# Kinetochores, cohesin, and DNA breaks: Controlling meiotic recombination within pericentromeres

**DOI:** 10.1002/yea.3366

**Published:** 2019-02-03

**Authors:** Lisa‐Marie Kuhl, Gerben Vader

**Affiliations:** ^1^ Department of Mechanistic Cell Biology Max Planck Institute of Molecular Physiology Dortmund Germany

**Keywords:** cohesin, DNA breaks, kinetochore, meiotic recombination, (peri)centromeres, *Saccharomyces*, *Schizosaccharomyces*

## Abstract

In meiosis, DNA break formation and repair are essential for the formation of crossovers between homologous chromosomes. Without crossover formation, faithful meiotic chromosome segregation and sexual reproduction cannot occur. Crossover formation is initiated by the programmed, meiosis‐specific introduction of numerous DNA double‐strand breaks, after which specific repair pathways promote recombination between homologous chromosomes. Despite its crucial nature, meiotic recombination is fraud with danger: When positioned or repaired inappropriately, DNA breaks can have catastrophic consequences on genome stability of the resulting gametes. As such, DNA break formation and repair needs to be carefully controlled. Within centromeres and surrounding regions (i.e., pericentromeres), meiotic crossover recombination is repressed in organisms ranging from yeast to humans, and a failure to do so is implicated in chromosome missegregation and developmental aneuploidy. (Peri)centromere sequence identity and organization diverge considerably across eukaryotes, yet suppression of meiotic DNA break formation and repair appear universal. Here, we discuss emerging work that has used budding and fission yeast systems to study the mechanisms underlying pericentromeric suppression of DNA break formation and repair. We particularly highlight a role for the kinetochore, a universally conserved, centromere‐associated structure essential for chromosome segregation, in suppressing (peri)centromeric DNA break formation and repair. We discuss the current understanding of kinetochore‐associated and chromosomal factors involved in this regulation and suggest future avenues of research.

## 1 MEIOSIS: DNA BREAKS, RECOMBINATION, AND SPECIALIZED CHROMOSOME SEGREGATION

**Figure 1:**
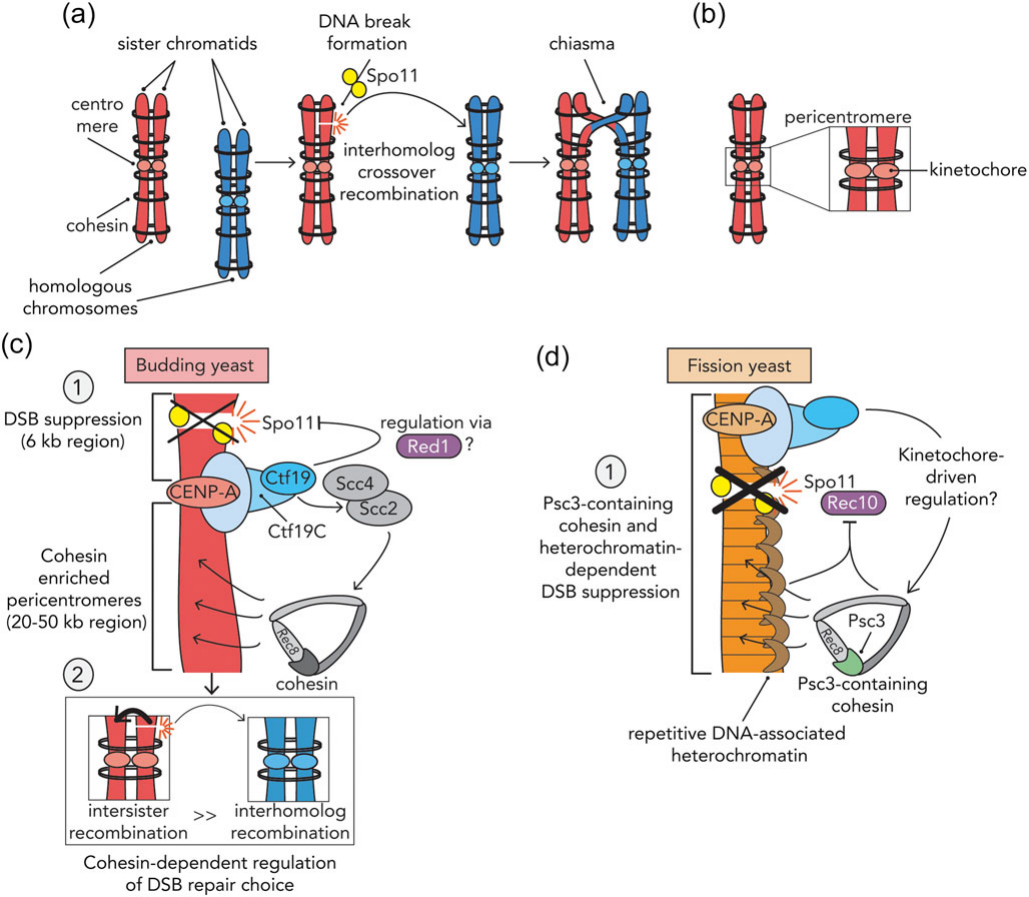
(a) Schematic of the processes that lead to the formation of physically linked homologous chromosomes. After Spo11‐dependent double‐strand break (DSB) formation, interhomolog crossover (CO) repair leads to the formation of a chiasma. (b) Schematic of a close up of the chromosomal region surrounding the centromere region of a chromosome (i.e., the pericentromere). The centromere is the genomic region where the kinetochore is nucleated. (c) DSB and CO regulation at budding yeast chromosomes. Emanating from kinetochores are two signal that lead to suppression of (1) Spo11‐dependent DSBs within ~6‐kb‐sized regions surrounding centromeres, and (2) Ctf19C‐dependent recruitment of pericentromeric cohesin is steering (residual) DSB formation into intersister‐directed repair instead of interhomolog‐directed repair. As such, CO formation is minimized in the entire pericentromeric regions (i.e., in ~20‐ to 50‐kb‐sized regions). (d) Within fission yeast pericentromeres, specialized cohesin (containing Psc3), in conjunction with heterochromatin, prevents the localization of Rec10, an essential factor required for Spo11‐dependent DSB formation. This leads to a suppression of DSBs and of CO formation.

The meiotic cell division program produces gametes containing one copy of each chromosome (i.e., these cells are haploid). Fusion of two gametes upon fertilization reconstitutes the diploid genome of the zygote. Meiosis relies on the same basic molecular machinery as mitosis but requires additional dedicated events (Petronczki, Siomos, & Nasmyth, 2003). Similar to mitosis, the meiotic program starts with DNA replication, which in meiosis is followed by two chromosome segregation phases instead of one. During the first chromosome segregation phase (i.e., meiosis I; MI), homologous chromosomes segregate. This is followed by meiosis II (MII), when sister chromatids segregate. In both cases, dynamic interactions between microtubules of the spindle apparatus and chromosomes drive meiotic chromosome segregation. These interactions are mediated by kinetochores, large chromatin‐associated protein assemblies that nucleate on genomic regions termed centromeres (Musacchio & Desai, 2017). Sister chromatids (and eventually also homologs; see below) are held together (i.e., cohered) by cohesin, a large ring‐shaped protein complex. Controlled cleavage of a cohesin subunit leads to loss of chromatid cohesion, and, consequently, homolog and chromatid disjunction in MI and MII, respectively. Faithful segregation of homologs in meiosis I requires physical connections between them (Petronczki et al., 2003). Such linkages are initially absent and are established de novo before meiosis I via the use of homologous recombination (HR)‐mediated repair (Keeney, 2001). HR can repair DNA double‐strand breaks (DSBs) by using DSB‐flanking sequences to identify a repair template elsewhere in the genome. Upon templating, DNA synthesis and re‐ligation coupled to controlled resolution of repair intermediates results in repair of the DSB lesion. In a diploid cell, allelic templates are found on the sister chromatid and the homologous chromosome. Repair outcome is determined by template usage (i.e., sister chromatid or homolog) and repair intermediate resolution. Repair using the homologous chromosome as a template can be resolved to result in the reciprocal exchange of flanking chromosomal arm regions, in what is known as a crossover (CO) repair product. A CO, in combination with cohesin present distally to the recombination site, forms what is called a chiasma and establishes a physical connection between homologs (Figure 1a). Alternatively, DSBs can be repaired without exchange of flanking regions, leading to a non‐CO (NCO). A collateral consequence of interhomolog (IH) recombination is the establishment of novel genetic combinations on individual chromosomes. Meiotic HR is initiated by DSBs that are introduced by a topoisomerase‐like enzyme called Spo11, together with several auxiliary proteins (together referred to as the meiotic DSB machinery; Keeney, 2001). Meiotic HR repair is normally biased towards the use of the homolog as repair template, which is essential to ensure CO formation (Humphryes & Hochwagen, 2014). For example, in budding yeast, every meiotic cell experiences ~140–170 DSBs (Pan et al., 2011). The amount of COs/cell ranges from 75 to 100, depending on the exact strain background that is used (Chen et al., 2008; Martini et al., 2011). The remainder of DSBs are repaired as NCOs, or via intersister (IS)‐directed HR. Although DSBs are required for gamete formation, aberrant DSB repair endangers genome stability (Sasaki, Lange, & Keeney, 2010). Notably, meiotic DSB formation and repair within specific regions of the genome (such as (peri)centromeres, repetitive DNA arrays and telomeres) has been associated with increased genome instability or chromosome missegregation (e.g., Hassold & Hunt, 2001; Koehler, Hawley, Sherman, & Hassold, 1996; Rockmill, Voelkel‐Meiman, & Roeder, 2006; Su, Barton, & Kaback, 2000; Vader et al., 2011). Thus, meiotic DSB formation and repair requires careful control. Spo11‐dependent DSBs are formed in a nonrandom fashion, with regions of high DSB activity (i.e., DSB hotspots) and regions of minimal DSB activity (i.e., DSB cold regions). Often, DSB cold regions fall within genomic regions that are vulnerable during DSB repair, and these cold regions likely reflect local control. DSB placement depends on multiple factors, such as nucleosome occupancy (i.e., Spo11 prefers to cleave within nucleosome depleted regions), chromatin modifications, and higher order chromosome organization (Pan et al., 2011). In addition to local regulation of DSB activity, the repair choice during HR (i.e., homolog vs. sister and/or CO vs. NCO) can also be subject to regulation. In conclusion, localized control systems act to protect at‐risk genomic regions against unwanted DSB formation and repair.

## 2 (PERI)CENTROMERES, KINETOCHORES, AND COHESIN

Kinetochores are nucleated onto specific genomic regions termed centromeres (Musacchio & Desai, 2017; Figure 1b). Between species, centromeres and (peri)centromeres (i.e., the genomic regions that directly surround centromeres) differ in sequence and organization (Allshire & Karpen, 2008; McKinley & Cheeseman, 2016). In budding yeast, centromeres are short (~125 base pair) sequences that are recognized by specific kinetochore proteins and bind a single nucleosome containing Cse4 (commonly referred to as CENP‐A), a centromere‐specific histone H3 variant (Hegemann & Fleig, 1993; Musacchio & Desai, 2017). Budding yeast pericentromeres are not defined by specific sequence and instead are very similar to genomic regions elsewhere in the yeast genome (i.e., they contain a normal density of active, Pol‐II‐transcribed genes). Many other eukaryotes, including fission yeast, have more complex pericentromere identities, where specific (often repetitive and non‐coding) sequences play a role in the establishment of defined chromatin environments (i.e., heterochromatic, transcriptionally silenced regions; Allshire & Karpen, 2008; Grewal & Jia, 2007). In addition, pericentromeres often contain specialized cohesin domains. This can be established via pericentromere‐specific enrichment of canonical cohesin levels (e.g., Rec8‐containing cohesin in budding yeast meiosis; Glynn et al., 2004; Tanaka, Cosma, Wirth, & Nasmyth, 1999; Weber et al., 2004) or via the recruitment of specialized cohesin complexes, like in fission yeast (Kitajima, Yokobayashi, Yamamoto, & Watanabe, 2003). In addition, fission yeast pericentromeres also contain increased density of cohesin, and enrichment is mediated by pericentromeric heterochromatin (Nonaka et al., 2002). Work from budding yeast has established a clear connection between kinetochore function and pericentromeric enrichment of cohesin (Fernius et al., 2013; Fernius & Marston, 2009; Weber et al., 2004). As such, at least when considering cohesin function, the kinetochore dictates local chromatin function and thus affects pericentromeric chromatin.

## 3 CONTROL OF MEIOTIC RECOMBINATION WITHIN PERICENTROMERES

Pericentromeres are regions of low meiotic DSB formation and CO formation (Blitzblau, Bell, Rodriguez, Bell, & Hochwagen, 2007; Borde, Wu, & Lichten, 1999; Buhler, Borde, & Lichten, 2007; Centola & Carbon, 1994; Copenhaver et al., 1999; Ellermeier et al., 2010; Gerton et al., 2000; Gore et al., 2009; Lambie & Roeder, 1988; Mahtani & Willard, 1998; Nakaseko, Adachi, Funahashi, Niwa, & Yanagida, 1986; Pan et al., 2011; Puechberty et al., 1999; Saintenac et al., 2009; Westphal & Reuter, 2002). In several species (including yeast and humans), (peri)centromeric CO formation is associated with increased meiotic chromosome missegregation that causes aneuploidy (Hassold & Hunt, 2001; Koehler et al., 1996; Rockmill et al., 2006). Pericentromeric COs can lead to chromosome missegregation through different paths. Disjunction of homologs requires timed removal (during MI) of cohesin laid down distally to a chiasma (i.e., a CO; Petronczki et al., 2003). As such, when a chiasma is established within a region where cohesin is protected against removal (as is the case for pericentromeric cohesin in MI), this will complicate disjunction of homologs. Thus, one possible outcome of chiasma establishment within a pericentromere is nondisjunction of homologs in meiosis I (Koehler et al., 1996; Lamb, Sherman, & Hassold, 2005). An opposite effect can also occur: CO formation within the region where cohesin needs to be maintained past meiosis I (in order to maintain sister chromatid cohesion until MII) can lead to a local weakening of cohesive forces, resulting in premature sister chromatid separation during meiosis I (Rockmill et al., 2006). Both these scenarios have been reported in different model systems, and their prevalence could be determined by organismal differences in cohesin levels and timing of cohesin removal. In any case, the placement of COs within pericentromeres can lower the fidelity of meiotic chromosome segregation.

Are there pericentromere‐specific DNA breakage and repair control systems to shield organisms against the dangers of genome instability and reduced reproductive fitness? Genome‐wide mapping studies of Spo11‐dependent DSB formation in budding yeast revealed a depression of DSB activity within a several kilobase (kb)‐sized regions directly adjacent to centromeres (Blitzblau et al., 2007; Buhler et al., 2007; Gerton et al., 2000; Pan et al., 2011). In addition, genome‐wide recombination mapping revealed regions surrounding centromeres that are reminiscent of the regions defined as pericentromeres, where CO formation is lower than the observed genome average (Chen et al., 2008; Mancera, Bourgon, Brozzi, Huber, & Steinmetz, 2008). It is important to note that pericentromeric DSB reduction in budding yeast is not absolute: DSBs can be formed within these regions, but to lower total levels as compared with genome‐wide DSB levels (Blitzblau et al., 2007; Buhler et al., 2007). Together with the observation that, despite residual DSB activity, CO and NCO formation is suppressed within pericentromeres (Chen et al., 2008); this suggests that DSB suppression alone cannot explain the observed suppression of COs within pericentromeres. Instead, it implies the existence of DSB repair pathway regulation within pericentromeres that disfavors CO formation over other types or repair outcomes. For example, at pericentromeres, HR‐driven repair of DSBs could preferentially yield IS‐directed repair over IH‐directed repair (Chen et al., 2008). This kind of IS > IH bias during repair contrasts with the IH > IS bias observed for canonical meiotic DSB repair (Humphryes & Hochwagen, 2014) and is more reminiscent of mitotic HR‐mediated repair of DSBs, when repair via sister chromatids is greatly preferred over IH repair. Work in budding yeast identified two factors (Zip1 and Sgs1) that influence pericentromeric CO formation, in a manner that is likely independent of DSB suppression (Chen et al., 2008; Rockmill et al., 2006). These factors thus act during the repair process after DSB formation. However, Zip1 and Sgs1 are not specific to (peri)centromeres, and it remained unclear how pericentromeres specifically affect meiotic DSB formation and recombination (Talbert & Henikoff, 2010). A study by Ellermeier and co‐workers focussed on the role of fission yeast heterochromatin function in controlling meiotic DSB formation and found that mutations that impair the proper establishment of pericentromeric heterochromatin (via mutations in the RNAi and the heterochromatin pathways) can relieve the suppression of recombination normally observed around fission yeast centromeres (Ellermeier et al., 2010). These mutations caused an increase in DSB formation at pericentromeres, demonstrating that specialized chromatin can influence DSB propensity. However, not every organism possesses centromeres that are embedded in specialized chromatin containing repetitive DNA and heterochromatin (see above), raising the question of how such pericentromeres might be protected. We recently addressed this question by focussing on budding yeast (Vincenten et al., 2015). By definition, the one unifying characteristic of all pericentromeres, regardless of underlying sequence, is chromosomal (and physical) proximity to centromeres and thus to kinetochores. We asked whether kinetochores influence meiotic DSB formation and recombination within budding yeast pericentromeres. Kinetochores are assembled on CENP‐A‐containing nucleosome via the cooperative binding of subcomplexes that execute kinetochore‐associated functions (e.g., microtubule binding and checkpoint signalling; Musacchio & Desai, 2017). One of these complexes is the CCAN (for Constitutive Centromere‐Associated Network; Cheeseman & Desai, 2008). In budding yeast, this complex is named the Ctf19‐complex (Ctf19C; Malvezzi & Westermann, 2014), and as its general name implies, the Ctf19C/CCAN is present at centromeres during all (mitotic and meiotic) cell cycle phases. Intriguingly, we found that the kinetochore (and the Ctf19C) plays an active role during both DSB formation and DNA repair choice at pericentromeres (Vincenten et al., 2015). Inactivating components of the Ctf19C triggers a strong and specific increase of recombination within pericentromeres (of ~21‐fold). Thus, the Ctf19C specifically influences pericentromeric recombination. The localization of the kinetochore (and the Ctf19C) is strictly limited to the single CENP‐A‐containing nucleosome assembled at centromeres and does not bind pericentromeric sequences (Pekgöz Altunkaya et al., 2016). In total, our findings demonstrated that the kinetochore (and the Ctf19C) affects meiotic DSB and repair control within pericentromeres “at a distance.”

How is the kinetochore able to do this? Experiments in both budding and fission yeasts have started to provide potential answers to this question. First, the Ctf9C affects local DSB formation at budding yeast pericentromeres (Figure 1c; Vincenten et al., 2015). It does so uniformly around all centromeres, and within a region of ~6 kb surrounding centromeres (i.e., within ~3 kb on both sides). In cells that lack Ctf19C components, a 5‐fold increase was detected in DSB formation. How the kinetochore (and the Ctf19C) affects local DSB activity is unclear, but we speculate it influences local chromosome and/or chromatin organization in order to affect Spo11 activity (see also below). The total increase in recombination frequencies observed in cells lacking Ctf19C components was larger than the observed effect on DSB activity only (~21‐fold vs. ~5‐fold), hinting at an additional Ctf19C‐derived effect that acts post‐DSB formation. Indeed, further analysis revealed that the Ctf19C reduces CO frequency, and it does so through its effect on pericentromeric cohesin enrichment (Figure 1c). The Ctf19C is directly involved in cohesin enrichment in mitotically dividing cells. Recruitment of a “cohesin loader” (containing the Scc2 and Scc4 proteins) to kinetochores is mediated via the Ctf19C (Fernius et al., 2013; Fernius & Marston, 2009). Specifically, within the Ctf19C, the Ctf19 protein directly recruits the Scc2/4 complex (Hinshaw, Makrantoni, Harrison, & Marston, 2017; Hinshaw, Makrantoni, Kerr, Marston, & Harrison, 2015). Local enrichment of Scc2/4 in turn leads to an enrichment of cohesin complexes in 20–50 kb surrounding the centromere (Fernius et al., 2013). In meiosis, the Ctf19C is equally involved in local (Rec8‐containing) cohesin enrichment at pericentromeres (Vincenten et al., 2015), presumably via similar mechansims. By using mutants in either cohesin or in the Scc2/4 cohesin loader (Hinshaw et al., 2015), we revealed that local cohesin enrichment and function is not required for local DSB suppression (Vincenten et al., 2015) but does influence repair choice within pericentromeres. Thus, within budding yeast pericentromeres, cohesin steers DSB repair away from meiotic IH CO formation. In mitotic cells, the levels of cohesin on chromosomes affects DSB repair choices (i.e., cohesin promotes IS‐directed repair; Covo, Westmoreland, Gordenin, & Resnick, 2010). Therefore, high levels of cohesin within pericentromeres favor repair of DSBs using sister chromatids instead of homologs. In essense, when considering repair of meiotically‐induced DSBs, pericentromeres behave more like mitotic than meiotic chromosomes. Interestingly, recent work in fission yeast confirmed on a connection between local cohesin function and control of meiotic recombination (Figure 1d; Nambiar & Smith, 2018). Within fission yeast pericentromeres, a specialized type of cohesin complex (containing Rec8 and Psc3) is present during meiosis, whereas within chromosome arms, cohesin complexes contain Rec8 and Rec11 (Kitajima et al., 2003). By using a variety of targeting systems to recruit cohesin complexes and meiotic DSB factors, Nambiar and Smith concluded that fission yeast pericentromeres are recombination coldspots because of the differential presence of Psc3‐, and not Rec11‐, containing cohesin complexes (Nambiar & Smith, 2018). In combination with pericentromeric heterochromatin, this difference leads to inhibition of Spo11‐dependent DSB formation and, consequently, CO formation. It is worth noting that according to this model, all regulation acts at the level of Spo11‐dependent DSB formation, without the need for additional regulation at the level of DSB repair choice. This is in contrast to budding yeast, where two layers of regulation exist, both at the level of DSB formation and at the level of repair choice (Vincenten et al., 2015). It will be interesting to further explore why distinct types of regulation might have evolved. One potential explanation is that, because of the repetitive nature of fission yeast pericentromeric sequences, sister chromatid‐directed repair can also endanger repeat stability (via nonallelic IS repair between identical sequences), thus necessitating complete shutdown of DSB formation.

How is DSB formation at (budding yeast or fission yeast) pericentromeres prevented? Ectopic targeting experiments by Robine et al. (2007) revealed that forcing Spo11 to pericentromeres was insufficient to induce DSB activity, hinting at regulation beyond Spo11 localization. Spo11 activity requires auxiliary “DSB‐factors” and meiotic‐specific reorganization of chromosomes to function (Lam & Keeney, 2014). This reorganization is driven by the assembly of chromosomal factors (called linear elements in fission yeast), which co‐localize with cohesin complexes (Panizza et al., 2011). One protein required for this assembly is a structural protein called Red1 (in budding yeast; the homolog of Red1 in fission yeast is called Rec10; Panizza et al., 2011; Sun et al., 2015). Work in fission yeast demonstrated that an interaction between Rec11‐containing cohesin and Rec10 is needed for the efficient recruitment of Rec10 to fission yeast chromosomes (Phadnis et al., 2015; Sakuno & Watanabe, 2015). Pericentromeric cohesin contains Psc3 instead of Rec11 (see above; Kitajima et al., 2003), leading to an absence of Rec10 within pericentromeres. Interestingly, forced recruitment of Rec10 showed that the absence of Rec10 at wild‐type pericentromeres is a determining step in controlling Spo11‐dependent DSB formation (Nambiar & Smith, 2018). These results suggest that, within fission yeast pericentromeres, specific recruitment of Psc3‐containing cohesin (in combination with pericentromeric heterochromatin), prevents the assembly of a “DSB‐permissive” chromatin environment, through exclusion of Rec10 (Figure 1d). Could a similar mechanism control DSB formation in budding yeast? Along budding yeast chromosomes, Red1 strongly co‐localizes with Rec8‐cohesin (Panizza et al., 2011; Sun et al., 2015). In addition, Red1 interacts with and requires Rec8‐cohesin for its normal chromosomal association (Panizza et al., 2011; Sun et al., 2015). From these data, it seems likely that an interaction between cohesin and Red1 is central to driving Red1 chromosomal loading (possibly via Scc3, the homolog of Psc3/Rec11, in a manner analogous to fission yeast Rec11‐Rec10 axis (Phadnis et al., 2015; Sakuno & Watanabe, 2015), although this interaction might also involve Rec8 directly (Sun et al., 2015). Interestingly, within budding yeast pericentromeres, Red1 and cohesin chromosomal patterns diverge: Here, Red1 levels are lower than cohesin levels (Sun et al., 2015). This hints at pericentromeric regulation of Red1 function and, potentially, DSB activity. Strikingly, the lower levels of Red1 at pericentromeres seem to be mediated via Hop1, a HORMA domain‐containing protein that is associated with Red1 and cohesin (Sun et al., 2015). It will be interesting to investigate whether inactivating kinetochore factors (e.g., Ctf19C) influences Red1 chromosomal localization.

Taken together, a localized regulation of Red1 function within budding yeast pericentromeres could play a role in controlling DSB formation, in a manner that might share foundational principles within the fission yeast system. However, clear differences between fission and budding yeast regulation of DSB formation and repair exist. Most importantly, whereas in fission yeast, DSB regulation involves cohesin (Nambiar & Smith, 2018), DSB control within budding yeast pericentromeres (even if putatively involving controlled Red1 recruitment) occurs independently of cohesin (Vincenten et al., 2015).

## 4 CONCLUSION AND OUTSTANDING QUESTIONS

As discussed here, fascinating new aspects of localized control of meiotic DSB formation and repair have recently been revealed. Despite structural differences within pericentromeres, a common thread is a key role for cohesin in controlling local DSB formation and repair (Nambiar & Smith, 2018; Vincenten et al., 2015). In one case (i.e., budding yeast), cohesin affects repair decisions after DSB have been formed, whereas in the other case (i.e., fission yeast), specialized cohesin complexes affect meiotic DSB formation. It will be interesting to establish whether, in the latter system, cohesin also affects repair decisions post‐DSB formation (in a manner analogous to budding yeast). It is clear that, in budding yeast, kinetochores are key orchestrators of pericentromeric control of DSB formation and recombination. Within kinetochores, the conserved Ctf19C influences both DSB suppression (in a currently unknown manner) and repair decisions (by directing local cohesin enrichment). How the Ctf19C controls DSB formation and whether this control also involves regulation at the level of Red1/Rec10 recruitment, as it is the case in fission yeast, are exciting future research questions. The fission yeast genome contains a complex that is homologous to the Ctf19C/CCAN (with, fta2p being the fission yeast homolog of the Ctf19 protein). It will be interesting to investigate whether and, if so, how kinetochores (and specifically the Ctf19C/CCAN) are involved in pericentromeric DSB suppression during meiosis in other species, including fission yeast. In any case, our findings in budding yeast have expanded the wide array of functions that have been ascribed to the kinetochore, and it will be exciting to deepen our understanding of the connections between kinetochores, cohesin, and pericentromeric control of meiotic DSB formation and repair.
